# Are FXR Family Proteins Integrators of Dopamine Signaling and Glutamatergic Neurotransmission in Mental Illnesses?

**DOI:** 10.3389/fnsyn.2018.00022

**Published:** 2018-07-24

**Authors:** Jivan Khlghatyan, Jean-Martin Beaulieu

**Affiliations:** ^1^Department of Pharmacology and Toxicology, Medical Sciences Building, University of Toronto, Toronto, ON, Canada; ^2^Department of Psychiatry and Neuroscience, Faculty of Medicine, Université Laval, Quebec, QC, Canada

**Keywords:** fragile X proteins, dopamine signaling, schizophrenia, mood disorders, glutamatergic neurotransmission

## Abstract

Dopamine receptors and related signaling pathways have long been implicated in pathophysiology and treatment of mental illnesses, including schizophrenia and bipolar disorder. Dopamine signaling may impact neuronal activity by modulation of glutamate neurotransmission. Recent evidence indicates a direct and/or indirect involvement of fragile X-related family proteins (FXR) in the regulation and mediation of dopamine receptor functions. FXRs consists of fragile X mental retardation protein 1 (Fmr1/FMRP) and its autosomal homologs Fxr1 and Fxr2. These RNA-binding proteins are enriched in the brain. Loss of function mutation in human *FMR1* is the major genetic contributor to Fragile X mental retardation syndrome. Therefore, the role of FXR proteins has mostly been studied in the context of autism spectrum disorders. However, recent genome-wide association studies have linked this family to schizophrenia, bipolar disorders, and mood regulation pointing toward a broader involvement in mental illnesses. FXR family proteins play an important role in the regulation of glutamate-mediated neuronal activity and plasticity. Here, we discuss the brain-specific functions of FXR family proteins by focusing on the regulation of dopamine receptor functions, ionotropic glutamate receptors-mediated synaptic plasticity and contribution to mental illnesses. Based on recent evidence, we propose that FXR proteins are potential integrators of dopamine signaling and ionotropic glutamate transmission.

## Introduction

The neurotransmitter dopamine is involved in the regulation of several behavioral dimensions including locomotion, reward, affect, mood, and cognitive functions. Dopamine exerts its actions by activating two groups of G protein couple receptors termed D1-class (D1 and D5) and D2-class (D2S, D2L, D3, and D4) (Missale et al., 1998; Beaulieu and Gainetdinov, 2011). D1-class receptors are positively coupled to adenylate cyclase to stimulate the production of the second messenger cAMP. In contrast, D2-class receptors inhibit cAMP production. The D2 dopamine receptor (D2R) also exerts its actions by engaging a beta-arrestin 2 (βArr2) mediated signaling pathway resulting in the inactivation of the protein kinase Akt and the concomitant activation of its substrates glycogen synthase kinase 3 alpha and beta (Gsk3α/β) (Beaulieu et al., 2004, 2005).

One major downstream consequence of dopamine receptor signaling is the regulation of ion channels and ionotropic receptors for neurotransmitters (Snyder et al., 2000; Svenningsson et al., 2004; Beaulieu and Gainetdinov, 2011; Devor et al., 2017). A great deal of attention has been given to the roles of cAMP and protein kinase A-mediated signaling in the regulation of ionotropic glutamate receptors α-amino-3-hydroxy-5-methyl-4-isoxazole propionic acid (AMPA) and *N*-methyl-D-aspartate (NMDA). Furthermore, dopamine receptor signaling has also been shown to regulate the expression of genes involved in ionotropic glutamate transmission (Yao et al., 2004; Renthal and Nestler, 2008; Renthal et al., 2008).

The fragile X mental retardation syndrome-related protein 1 (Fxr1), Fxr2, and fragile X mental retardation protein 1 (Fmr1) comprise a small family of RNA binding proteins named fragile X-related (FXR) proteins. Mutations in Fmr1 gene are linked to human fragile X syndrome and autism (Crawford et al., 2001). However, recent genetic studies linked FXR family to schizophrenia, bipolar disorders, and mood regulation, indicating their wider role in adult-onset mental disorders (Bowden et al., 2008; Fromer et al., 2014; Purcell et al., 2014; Schizophrenia Working Group of the Psychiatric Genomics Consortium, 2014; Takata et al., 2017). Dopamine-mediated signaling is impaired in many of these pathologies (Howes and Kapur, 2009; Ashok et al., 2017). In addition, recent studies point toward possible direct or indirect implications of FXR family proteins in dopamine receptor regulation and signaling. Here, we explore the possible contribution of FXR proteins to the integration of dopamine and ionotropic receptor-mediated glutamate signaling in the broader context of adult onset mental disorders and addiction.

## FXR Proteins in the Brain

The FXR family proteins are enriched in the brain. Immunolabeling revealed widespread expression of Fxr1, Fxr2, and Fmr1 in fetal and adult human and mouse brains. These proteins are mostly localized to the cytoplasm and proximal dendrites of neurons. In addition, the Fxr1 expression is also found in the nucleus of neurons in the fetal human brain. Very limited expression of Fxr1 was observed in glia by immunostaining (Tamanini et al., 1997; Bakker et al., 2000; Cook et al., 2011). However, recent investigations using RNA-Seq identified transcripts of Fxr1, Fxr2, and Fmr1 in different cell types of the mouse cortex. In contrast to the Fmr1 transcript, which is mostly present in neurons and astrocytes, Fxr1 and Fxr2 transcripts have also been detected in oligodendrocytes, microglia, and endothelial cells in the cortex of mice (Thomsen et al., 2013; Zhang et al., 2014).

Investigation of subcellular localization by immunoelectron microscopy and biochemical methods showed that all three FXR proteins are associated with polyribosomes in the cytoplasm and along the dendrites of neurons (Tamanini et al., 1997; Bakker et al., 2000; Darnell et al., 2009; Cook et al., 2011). All FXRs are detected in mouse brain synaptosomes (Tamanini et al., 1997; Cook et al., 2011). Fxr1 and Fmr1 are observed in the base of dendritic filopodia and spines in proximal and distal dendrites of hippocampal cultures (Antar et al., 2005; Cook et al., 2011). Subsets of dendritic Fxr1 clusters also contain Fxr2 and Fmr1 (Cook et al., 2011). Fxr1, Fxr2, and Fmr1 are enriched in RNA granules (stalled ribosomes) in the developing mouse brain. Interestingly, all FXR proteins are co-detected only in 22% of RNA granules, the rest of the granules have different combinations of two or only one FXR protein, indicating possible overlapping and nonoverlapping functions of FXR proteins in RNA granules (El Fatimy et al., 2016).

The FXR family proteins are also found in presynaptic compartments in so-called presynaptic fragile X granules (FXGs). Despite the presence of FXR proteins in the somatodendritic compartment of virtually every neuron, their presynaptic localization is more restricted. FXGs are detected in a subset of neurons in frontal cortical, hippocampal area CA3, and olfactory bulb glomeruli, only in developing mouse brains. Studies of granule composition showed that all three FXR family proteins were present in cortical FXGs, while the majority of granules in the hippocampus and olfactory glomeruli only contained Fxr2 and Fmr1. Knockout mouse studies demonstrated that Fxr2 is crucial for formation and Fmr1 for regulation of FXGs (Christie et al., 2009). Overall, expression and subcellular localization data suggest that FXR proteins can have shares as well as exclusive brain functions.

## FXR Proteins Are Regulators of Ionotropic Glutamate Receptor-Mediated Synaptic Plasticity

The contribution of Fmr1 and Fxr2 to the regulation of metabotropic glutamate receptor functions has been studied extensively. For example, Fxr2 KO mice exhibit a reduction in metabotropic glutamate receptor-dependent long-term depression (mGluR-LTD), while the same type of plasticity is enhanced in Fmr1 and Fxr2/Fmr1 double knockout mice. Further characterization of underlying mechanisms pointed toward a dependence of mGluR-LTD on protein synthesis in Fxr2 KO mice, while it would be only partially dependent upon protein synthesis in Fxr2/Fmr1 double KO mice (Zhang et al., 2009).

**FIGURE 1:**
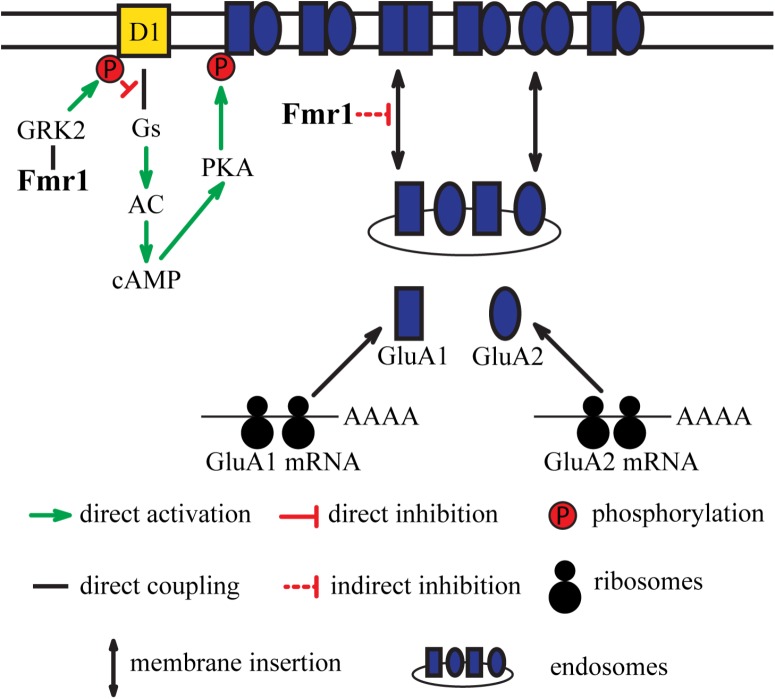
Integration of dopamine receptor D1-mediated signaling and glutamatergic neurotransmission by Fmr1. D1 receptor-mediated signaling results in phosphorylation of GluA1 subunits of AMPA receptors. Fmr1 regulates phosphorylation of D1 receptors via direct interaction with GRK2. This regulation of D1 by Fmr1 subsequently impacts phosphorylation of GluA1. Fmr1 also regulates trafficking of GluA1 via unknown mechanism.

However, there is also evidence for an implication of these proteins in the regulation of ionotropic glutamate receptor functions. Fxr2 binds to the GluA1 mRNA coding sequence, stabilizing this transcript and regulating GluA1 expression. In line with this, Fxr2 also affected GluA1-mediated currents and surface availability. Fmr1 deficiency also results in decreased GluA1. However, this effect would not be mediated by a regulation of GluA1 synthesis, but a contribution of Fmr1 to GluA1 membrane expression (**Figure 1**). From a functional point of view, young mice lacking Fmr1 or both Fxr2 and Fmr1 display altered basal transmission, paired-pulse facilitation, and protein synthesis-dependent late-phase LTP (L-LTP). However, these parameters are unaffected at all ages in Fxr2 KO mice.

The neuronal functions and the regulation of glutamate neurotransmission by Fxr1 have been understudied as compared to other FXR proteins. Conditional knockout (cKO) of Fxr1 specifically from excitatory neurons of the forebrain resulted in a slight decrease in density and length of spines in hippocampal area CA1 cells (Cook et al., 2014). While normal basal synaptic strength, short-term plasticity, and protein synthesis-independent early phase long-term plasticity (E-LTP) were unaltered. However, Fxr1 cKO mice exhibit a significant increase in L-LTP compared to controls. This effect would be mostly due to an increase in AMPA receptor subunit GluA2, while NMDA receptors would be unaffected. Further analysis suggested that Fxr1 can directly regulate GluA2 by binding to a GU-rich element in the 5′ untranslated region (UTR) of GluA2 mRNA (Cook et al., 2014).

In contrast, adeno-associated virus (AAV)-mediated augmentation of Fxr1 levels in the adult medial prefrontal cortex (mPFC) resulted in a decrease of excitatory postsynaptic currents (EPSCs) amplitude and frequency. A similar effect was observed as a result of a CRISPR/Cas9-mediated reduction of Gsk3β expression in mPFC (Khlghatyan et al., 2018). Gsk3β can directly phosphorylate Fxr1 and negatively regulate its expression (Del’Guidice et al., 2015). Thus, modulation of EPSCs by Fxr1 can occur in response to its regulation by Gsk3β. Further investigations revealed a decrease in synaptic AMPA receptors along with a possible switch from predominantly heteromeric GluA1/GluA2 containing to homomeric GluA1 AMPA receptors (Khlghatyan et al., 2018).

## A Role for Fmr1 in Dopamine D1 Receptor Signaling and Regulation

Evidence exists for the functional interplay between the D1 receptor and Fmr1. Stimulation of D1 has been shown to cause dynamic changes in phosphorylation of Fmr1 and result in a regulation of translation of its target proteins (Wang et al., 2010). Stimulation of these receptors by agonists also increases surface expression of the GluA1 subunit. The effect of D1 agonists on surface GluA1 expression is abolished in Fmr1 KO mice. Additionally, electrophysiological recordings of mEPSCs showed that Fmr1 is necessary for D1 receptor-mediated synaptic integration of GluA1 subunit. Moreover, D1 receptor-mediated facilitation of LTP in the cingulate cortex is altered in Fmr1 KO mice, indicating the contribution of Fmr1 in D1 receptor-mediated synaptic plasticity (**Figure 1**).

Further studies revealed that D1 receptor is also hyperphosphorylated in the PFC of Fmr1 KO mice, which results in impaired coupling to Gαs proteins. G protein-coupled receptor kinases (GRKs) are known to phosphorylate dopamine receptors and regulate their binding to G proteins and beta-arrestins. Two classes of GRKs, GRK2 class (GRK2 and GRK3), and GRK4 class (GRK5 and GRK6) have been shown to differentially regulate D1 and D2 receptors (Gurevich et al., 2016). The absence of Fmr1 caused a redistribution of GRK2 to the surface that resulted in hyperphosphorylation of D1 receptors by GRK2 (**Figure 1**). No changes in GRK4 localization were observed in Fmr1 KO mice (Wang et al., 2008). However, it is possible that interaction between other FXR proteins and GRKs may exist and affect dopamine receptor functions. Notably, alterations of GRK6 levels have been shown to specifically affect D2 receptors mediated signaling (Gainetdinov et al., 2003; Gurevich et al., 2016). It may thus be of interest to investigate if an alteration in FXR proteins functions may also affect GRK6 and D2 mediated signaling.

## Does Fxr1 Play a Role in Dopamine D2 Receptor Beta-Arrestin-2-Mediated Signaling?

New evidence also suggests that Fxr1 may play a role in the regulation of ionotropic glutamate receptors by the D2 dopamine receptor. Dopamine regulates Gsk3β activity downstream of D2 (Beaulieu et al., 2004, 2005). Activation of D2 results in the in dephosphorylation and inactivation of Akt following the formation of a protein complex comprised of β-Arrestin-2 (βArr2), protein phosphatase 2A (PP2A) and Akt. This, inhibition of Akt by the D2 receptor prevents the inhibitory phosphorylation of Gsk3β by Akt and results in Gsk3β activation (Beaulieu et al., 2005). The βArr2/PP2A/Akt complex can be disrupted by lithium, resulting in an activation of Akt and subsequent phosphorylation and inactivation of Gsk3β (Beaulieu et al., 2008).

In addition to lithium, other mood stabilizers, such as lamotrigine and valproate can also indirectly inhibit brain Gsk3β. This inhibitory effect is absent in D2 KO and βArr2 KO mice, indicating possible engagement of D2R-βArr2-PP2A-Akt pathway (Del’ Guidice and Beaulieu, 2015). Chronic treatment with lithium, lamotrigine, and valproate increases Fxr1 expression in the striatum and prefrontal cortex. An effect that is absent in βArr2 KO mice (Del’Guidice et al., 2015) thus suggesting an engagement of D2R-βArr2-Gsk3 pathway in the regulation of Fxr1 levels by these drugs.

**FIGURE 2:**
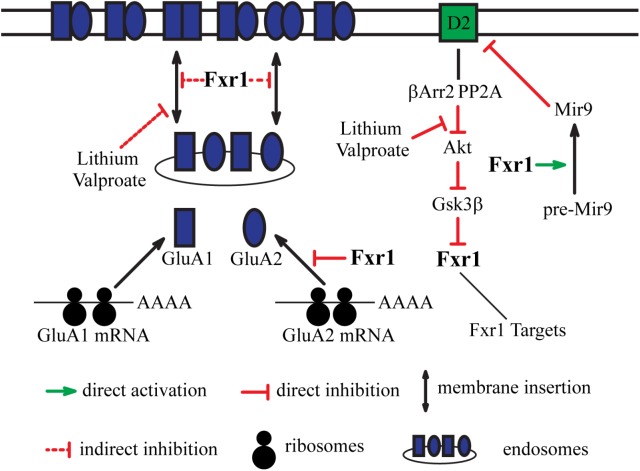
Integration of dopamine receptor D2-mediated signaling and glutamatergic neurotransmission by Fxr1. Fxr1 regulates trafficking of GluA1, GluA2, and synthesis of GluA2 subunits of AMPA receptors. Lithium and valproate regulate Fxr1 levels downstream of D2-βArr2-Akt-Gsk3β pathway. Lithium and valproate also regulate trafficking of GluA1, suggesting possible mechanistic involvement of Fxr1. Fxr1 regulates biogenesis of schizophrenia related Mir9, which, in turn, negatively regulates translation of D2 receptors.

Reduction of Gsk3β and augmentation of Fxr1 in mPFC caused a decrease in synaptic GluA1 and GluA2 subunits of AMPA receptors without affecting their synaptosomal expression levels. This indicates that the Gsk3β-Fxr1 pathway may be involved in regulation of the synaptic delivery of GluA1 and GluA2 (**Figure 2**) (Khlghatyan et al., 2018). Interestingly, chronic treatment with lithium or valproate reduces synaptosomal GluA1 levels and surface GluA1 distribution (Du et al., 2004). This effect is similar to what was observed following augmentation of Fxr1 or reduction of Gsk3β levels in the mouse mPFC (Khlghatyan et al., 2018). This suggests a contribution of the βArr2-Gsk3-Fxr1 pathway in the effect of lithium and valproate on surface GluA1 (**Figure 2**). A regulation of Fxr1 by βArr2-mediated D2 signaling would also provide a mechanism by which this receptor may regulate local protein synthesis and AMPA neurotransmission. However, while these observations are tantalizing the direct regulation of Fxr1 and GluA1 by D2 receptors still has to be further established by more investigations.

## FXR Proteins in Regulation of Dopamine-Associated Behaviors

Direct evidence for the contribution of dopamine in FXR proteins-mediated behaviors or the role of FXR proteins in dopamine-related behaviors remains scarce. However, there is evidence for an involvement of FXR proteins in the regulation of several behavioral modalities such as locomotion, gait, and sensory-motor gating, which are also regulated by dopamine neurotransmission.

Fxr2 KO mice exhibit hyperactivity in the open-field test, abnormal sensorimotor gating (acoustic startle response), impaired motor coordination, decreased response to heat stimulus, and impaired learning and memory performance in both contextual fear conditioning and the Morris water tasks (Bontekoe et al., 2002; Guo et al., 2015). Interestingly, Fmr1 KO mice exhibited several behavioral phenotypes similar to those of Fxr2 KO, such as hyperactivity and acoustic startle response impairment (Bontekoe et al., 2002; Spencer et al., 2006). Furthermore, some phenotypes like hyperactivity, sensory motor gating deficits, and fear conditioning are more pronounced in Fxr2/Fmr1 KO mice, indicating possible additive effects in specific behavioral paradigms (Bontekoe et al., 2002; Spencer et al., 2006). Interestingly, several behavioral phenotypes, such as hyperactivity of Fxr2 KO and Fmr1 KO mice and anxiety of Fmr1 KO mice were not replicated across studies (Spencer et al., 2006). A possible explanation for these discrepancies is that different genetic background and housing conditions can affect the penetrance of phenotypes associated to the deletion of these genes.

Stimulation of D1 receptor by agonists ameliorates hyperactivity phenotypes and motor coordination impairments in Fmr1 KO mice (Wang et al., 2008). Treatment with amphetamine also improved impaired novel object recognition in these mice (Ventura et al., 2004). In contrast, inhibition of Gsk3β, which can be activated by D2 receptors, results in reduced hyperactivity, improved cognition, and social behavior in this same mouse model (Yuskaitis et al., 2010; Mines and Jope, 2011).

There is also evidence for a contribution of Fmr1 in the responsiveness to psychostimulant drugs, which exert their effects by increasing dopamine extracellular tones (Beaulieu and Gainetdinov, 2011). Expression of Fmr1 in the medial prefrontal cortex has been found to be reduced in adult rats that were exposed to cocaine during adolescence (Caffino et al., 2014) and a mutagenesis study identified the cytoplasmic FMRP interacting protein 2 as a modulator of acute and sensitized locomotor responses to both cocaine and methamphetamine (Kumar et al., 2013). In line with this, Fmr1 KO mice display altered behavioral sensitization and reward to cocaine (Smith et al., 2014). This effect appears to involve different functions of this protein in different brain regions. On the one hand, behavioral sensitization would require Fmr1 expression in the adult *nucleus accumbens*, a region of the striatum that is involved in addiction-related behaviors. On the other hand, the regulation of mGluR5 by Fmr1, possibly in multiple brain regions would explain the contribution of Fmr1 to cocaine-associated reward (Smith et al., 2014).

Fxr1 KO mice die shortly after birth from cardiac and respiratory failure, thus behavioral testing is only possible after brain-specific alterations of Fxr1 expression (Mientjes et al., 2004). Fxr1 cKO has been achieved in mouse forebrain pyramidal neurons using a CamKII-Cre system and Fxr1 floxed alleles (Cook et al., 2014). These mice show no deficits in basic motor/sensory function, anxiety, contextual fear memory, and working memory. However, forebrain Fxr1 cKO mice display enhanced ability to recall spatial memories in a modified Morris water maze paradigm. In addition, these mice fail to shift their preference to the new platform location during the reversal probe test, thus demonstrating altered behavioral flexibility.

In contrast to gene inactivation, Fxr1 AAV-mediated overexpression in mPFC neurons of adult mice affects anxiety-related behaviors (Del’Guidice et al., 2015; Khlghatyan et al., 2018). A similar behavioral signature was observed after reduction of Gsk3β levels in forebrain pyramidal neurons in Gsk3β cKO mice (Latapy et al., 2012) and as a result of more specific AAV-mediated gene inactivation in the mPFC (Del’Guidice et al., 2015; Khlghatyan et al., 2018). In line with this, the interaction between Fxr1 and Gsk3β has been shown to affect mood and emotional processing in healthy humans (Del’Guidice et al., 2015).

The difference between cKO of Fxr1 and region-specific viral vector-mediated manipulations points to a role for Fxr1 in the regulation of different behaviors in a brain region-specific manner. This also suggests that functional impact of alterations in Fxr1 levels may depend on developmental stage. Finally, development of new approaches to manipulate Fxr1 expression in specific populations of dopamine receptor expressing cortical neurons will be needed to further characterize the contribution of dopamine neurotransmission in these behavioral phenotypes.

## FXR Proteins, Adult Onset Psychiatric Disorder Association

Loss of function of Fmr1 is one of the leading genetic causes of Fragile X and autism (Crawford et al., 2001). Structural similarities between FXR family proteins led to an investigation of Fxr1 and Fxr2 functions in the context of autism and fragile X syndrome. However, accumulation model of single nucleotide polymorphisms (SNPs) in FXR genes showed association with autistic traits not only in autistic patients but also in schizophrenics, patients with other neuropsychiatric disorders and healthy individuals, thus indicating a possible broader role of FXR genes (Stepniak et al., 2015). Indeed, recent human genetic and functional studies demonstrated the association of FXR genes and proteins to schizophrenia, bipolar disorders, and mood regulation.

Genome-wide association studies (GWAS) have linked SNPs in the *FXR1* locus to schizophrenia (Schizophrenia Working Group of the Psychiatric Genomics Consortium, 2014). Later, a GWAS associated SNP in this locus was shown to be in linkage disequilibrium with splicing quantitative trait loci (sQTL) SNPs identified in the human dorsolateral prefrontal cortex (DLPFC). This suggests that a schizophrenia-associated SNP in *FXR1* locus may possibly contribute to the disease by affecting alternative splicing (Takata et al., 2017). Mapping out potential schizophrenia networks by correlated expression of 369 genes from 108 schizophrenia-associated loci resulted in the identification of several pathways. The pathway with the largest number of correlations included 56 genes along with *DRD2* and *FXR1* (Liedtke et al., 2017). Alterations in microRNAs also have been linked to schizophrenia. Interestingly, schizophrenia-related genes are more likely to be regulated by microRNAs and Mir9 targetome is the most enriched in schizophrenia risk genes. Fxr1 have been shown to regulate the processing of several brain-specific microRNAs, including Mir9 (Xu et al., 2011). In turn, Mir9 targets and negatively regulates *DRD2* transcripts via binding to their 3′UTR region (Shi et al., 2014; Hauberg et al., 2016). Additionally, the micro-RNA that is the most significantly associated to schizophrenia Mir137 has been shown to inhibit the expression of the Fxr1 negative regulator Gsk3β by acting on the 3′UTR of the *GSK3B* mRNA (Thomas et al., 2017). This makes a strong case for the association of Fxr1 to schizophrenia and D2-mediated signaling (**Figure 2**). A possible contribution to the disease may be via alterations in splicing of Fxr1 with subsequent changes in the regulation of microRNAs and their targets such as the D2 receptor mRNAs.

Polymorphisms in the Fxr2 and Fmr1 loci have not been associated with schizophrenia by GWAS. However, some studies pointed to possible indirect contributions. Fxr2 levels have been shown to be altered in a small group of schizophrenia patient’s brain (Bowden et al., 2008). Furthermore, genome-wide epigenetic analysis conducted in monozygotic twins identified significant changes in methylation profile of Fxr2 gene in schizophrenic patients compared to healthy subjects (Dempster et al., 2011). Several studies identified changes in Fmr1 protein levels in brains of schizophrenic patients (Fatemi and Folsom, 2011). Decreased levels of Fmr1 may be associated with lower IQ and earlier onset of the disease (Kovács et al., 2013). Furthermore, recent large-scale studies indicated enrichment of Fmr1 targets in the genetic architecture of schizophrenia (Fromer et al., 2014; Purcell et al., 2014).

In addition to schizophrenia, some studies indicated that Fmr1 targets are enriched within genes that are shown to be affected in bipolar patients (Folsom et al., 2015; Goes et al., 2016). Furthermore, GWAS studies have identified SNPs in *FXR1* gene that shows significant association to bipolar disorder and comorbid eating disorder. Interestingly, anxiety spectrum disorders were co-occurring with a much higher rate in bipolar patients with an eating disorder (Liu et al., 2016). Thus, indicating the possible broad involvement of Fxr1 in the regulation of mood. Indeed, an interaction between functional polymorphisms in *FXR1* and *GSK3B* contributes to mood regulation in healthy subjects. Interestingly, the higher FXR1 expression is associated with greater emotional stability, except in the context of higher GSK3β expression (Del’Guidice et al., 2015). Gsk3β is a target of mood stabilizers and may contribute to the action of antipsychotics (Beaulieu et al., 2009). In line with this, animal studies demonstrated that Gsk3β can directly regulate Fxr1 levels in response to mood stabilizers and that increase in Fxr1 expression results in a decrease in anxiety-related behaviors (Del’Guidice et al., 2015; Khlghatyan et al., 2018). An interaction between *GSK3B* and *FXR1* functional polymorphisms was also found in a bipolar patient cohort, where it influenced the severity of manic and depressive symptoms (Bureau et al., 2017).

## Conclusion

Overall, several lines of evidence point toward the association of FXR family with neuropsychiatric disorders and its direct or indirect contribution to dopamine receptor signaling. While FXR proteins do not perform redundant functions, they may exert action on common targets like GluA1 and GluA2 via distinct mechanisms. Moreover, engagement of one or the other FXR protein can be a result of different upstream events. Thus, it is possible that FXR proteins may integrate the action of upstream players, such as D1 and D2 dopamine receptors, on common downstream targets, such as GluA1 and GluA2, via distinct mechanisms (**Figures 1**, **2**). Precise investigation of a role of FXR genes has to be carried in animal models in brain region and cell type-specific manner with consideration of developmental stages. Moreover, human functional imaging and genetic studies have to be carried out in context of interaction between FXR genes and their regulators and targets. Investigation of the potential role of FXR family proteins in dopamine signaling may lead to deeper understanding of underpinnings of mental disorders such as schizophrenia and mood disorders and may open new avenues for therapeutic development.

## Author Contributions

JK and J-MB contributed to the redaction of this manuscript.

## Conflict of Interest Statement

The authors declare that the research was conducted in the absence of any commercial or financial relationships that could be construed as a potential conflict of interest.
